# Supplementary material for: A clinical prediction model to differentiate tuberculous spondylodiscitis from pyogenic spontaneous spondylodiscitis
Source: PLoS One. 2023 Aug 18;18(8):e0290361. doi: 10.1371/journal.pone.0290361 (PMC10437852; doi:10.1371/journal.pone.0290361)
Supplement: S1 Checklist — (DOCX) [file pone.0290361.s001.docx]

STROBE Statement—checklist of items that should be included in reports of observational studies

|  | Item No. | Recommendation | Page  No. | Relevant text from manuscript |
| --- | --- | --- | --- | --- |
| **Title and abstract** | 1 | (*a*) Indicate the study’s design with a commonly used term in the title or the abstract | 2 | A retrospective single-center study |
|  |  | (*b*) Provide in the abstract an informative and balanced summary of what was done and what was found | 3 | This prediction model incorporating WBC, neutrophil fraction counts, ESR and presence of paraspinal abscess accurately predicted the causative pathogens. |
| Introduction | | | |  |
| Background/rationale | 2 | Explain the scientific background and rationale for the investigation being reported | 3 | Differentiating TS from PS is essential for appropriate management. However, diagnosing these two entities is challenging because of nonspecific signs and symptoms. Microbiological diagnosis is the benchmark for differentiating TS from PS, but determination of microorganism is difficult. |
| Objectives | 3 | State specific objectives, including any prespecified hypotheses | 4 | identify the predictive factors for differentiating TS from PS using clinical characteristics,… |
| Methods | | | |  |
| Study design | 4 | Present key elements of study design early in the paper | 4 | retrospective comparative study included patients with a diagnosis of SIS |
| Setting | 5 | Describe the setting, locations, and relevant dates, including periods of recruitment, exposure, follow-up, and data collection | 4 | This single-institution retrospective comparative study included patients with a diagnosis of SIS between January 2008 and December 2021 |
| Participants | 6 | (*a*) *Cohort study*—Give the eligibility criteria, and the sources and methods of selection of participants. Describe methods of follow-up  *Case-control study*—Give the eligibility criteria, and the sources and methods of case ascertainment and control selection. Give the rationale for the choice of cases and controls  *Cross-sectional study*—Give the eligibility criteria, and the sources and methods of selection of participants | 4 | The medical records of the adult patients (age ≥ 15 years) diagnosed with the first episode of either PS or TS and followed to the end of treatment were reviewed. |
|  |  | (*b*) *Cohort study*—For matched studies, give matching criteria and number of exposed and unexposed  *Case-control study*—For matched studies, give matching criteria and the number of controls per case | N/A |  |
| Variables | 7 | Clearly define all outcomes, exposures, predictors, potential confounders, and effect modifiers. Give diagnostic criteria, if applicable | N/A |  |
| Data sources/ measurement | 8* | For each variable of interest, give sources of data and details of methods of assessment (measurement). Describe comparability of assessment methods if there is more than one group | 4,5 | PS was diagnosed based on one of following criteria: (1) causative organism was isolated from spinal tissue at the suspected site of infection or (2) the patient had compatible spondylodiscitis signs and symptoms with radiological evidence of vertebral infection and positive blood cultures. TS was diagnosed based on one of following criteria: (1) histopathological findings established granuloma formation or positive polymerase chain reaction (PCR) for mycobacterium tuberculosis (M-TB) or (2) spinal tissue culture returned positive for M-TB. |
| Bias | 9 | Describe any efforts to address potential sources of bias | 5,6 | The X-rays and MRIs were independently evaluated by two expert musculoskeletal radiologists who were unaware of definitive diagnosis. If findings were discordant, the decision were made by consensus… |
| Study size | 10 | Explain how the study size was arrived at | 7 | During study period, 141 patients treated for PS or TS at our institution who met the criteria were enrolled in the study |

Continued on next page

| Quantitative variables | 11 | Explain how quantitative variables were handled in the analyses. If applicable, describe which groupings were chosen and why | 5 | Material and Methods – Data collection |
| --- | --- | --- | --- | --- |
| Statistical methods | 12 | (*a*) Describe all statistical methods, including those used to control for confounding | 6 | Independent t test (or quantile regression) and chi-square test (or exact test) were used to compare factors between TS and PS groups for continuous and categorical data, respectively….  The receiver operating characteristic (ROC) and Youden index analysis were used to determine the optimal biomarker cutoff values….  the multivariate logistic model. Forward selection was used for model selection. The performances of the final model were assessed by Hosmer-Lemeshow goodness-of-fit statistic…  Score performances—that is, the sensitivity, specificity, and positive and negative likelihood ratios (LR+/LR-)—were calculated according to each possible total score…. |
|  |  | (*b*) Describe any methods used to examine subgroups and interactions |  | N/A |
|  |  | (*c*) Explain how missing data were addressed |  | No data was missing |
|  |  | (*d*) *Cohort study*—If applicable, explain how loss to follow-up was addressed  *Case-control study*—If applicable, explain how matching of cases and controls was addressed  *Cross-sectional study*—If applicable, describe analytical methods taking account of sampling strategy |  | N/A |
|  |  | (*e*) Describe any sensitivity analyses |  | N/A |
| Results | | | | |
| Participants | 13* | (a) Report numbers of individuals at each stage of study—eg numbers potentially eligible, examined for eligibility, confirmed eligible, included in the study, completing follow-up, and analysed | Figure 1 | 168 patients diagnosed with spondylodiscitis; 16 patients were excluded (12 iatrogenic, 4 no definite diagnosis). Remaining 153 patients diagnosed with SIS; 12 patients were excluded…. |
|  |  | (b) Give reasons for non-participation at each stage |  | N/A |
|  |  | (c) Consider use of a flow diagram |  | Figure 1 |
| Descriptive data | 14* | (a) Give characteristics of study participants (eg demographic, clinical, social) and information on exposures and potential confounders | 8-10 | The demographic data, clinical characteristics and radiographic finding are shown in Table 1-3 |
|  |  | (b) Indicate number of participants with missing data for each variable of interest |  | None |
|  |  | (c) *Cohort study*—Summarise follow-up time (eg, average and total amount) |  | N/A |
| Outcome data | 15* | *Cohort study*—Report numbers of outcome events or summary measures over time |  | N/A |
|  |  | *Case-control study—*Report numbers in each exposure category, or summary measures of exposure |  | N/A |
|  |  | *Cross-sectional study—*Report numbers of outcome events or summary measures | 8-10    Figure 2 | The statistical difference of variables between PS and TS are shown in Table 1-3  Cut off value of biomarkers was illustrated in figure 2 (ROC analysis) |
| Main results | 16 | (*a*) Give unadjusted estimates and, if applicable, confounder-adjusted estimates and their precision (eg, 95% confidence interval). Make clear which confounders were adjusted for and why they were included | 13 | Table 5 showed Multivariate analysis of factors as an independent Predictors of TS |
|  |  | (*b*) Report category boundaries when continuous variables were categorized | 14 | Table 6 demonstrated scoring scheme of Prediction model to differentiate TS from PS |
|  |  | (*c*) If relevant, consider translating estimates of relative risk into absolute risk for a meaningful time period |  | N/A |

Continued on next page

| Other analyses | 17 | Report other analyses done—eg analyses of subgroups and interactions, and sensitivity analyses | 14 | low (score < 13), moderate (score between 13 and 19) and high (score ≥ 19) probability; with TS prevalence of 8.1%, 29.6% and 82.2%, respectively |
| --- | --- | --- | --- | --- |
| Discussion | | | | |
| Key results | 18 | Summarise key results with reference to study objectives | 16 | The present study identified four predictive factors—ESR, presence of paraspinal abscess on MRI, neutrophil fraction, and WBC count—as being helpful to distinguish TS from PS (Table 5) with excellent predictability (AUC = 0.921). This model was converted to a scoring scheme that justified an increased probability of TS among SIS patients (Table 6). |
| Limitations | 19 | Discuss limitations of the study, taking into account sources of potential bias or imprecision. Discuss both direction and magnitude of any potential bias | 18 | Single-center retrospective analysis. Second, study center is a referral center, which may have skewed the clinical or laboratory data regarding the onset of symptoms. Third, this study comprised by data for two cohorts collected in different period: year 2016 (January 2008 to December 2016 and year 2021 (January 2017 to December 2021). This approach may have involved bias owing to trends in spondylodiscitis management over the 13-year period. |
| Interpretation | 20 | Give a cautious overall interpretation of results considering objectives, limitations, multiplicity of analyses, results from similar studies, and other relevant evidence | 19 | the clinical presentations of other low virulent causative organisms, i.e. mycobacterium other than TB, fungi or brucellosis, may mimic TS which need to be clarified in future studies |
| Generalisability | 21 | Discuss the generalisability (external validity) of the study results | 19 | However, external validity of this scoring scheme still needs to be confirmed. |
| Other information | |  | | |
| Funding | 22 | Give the source of funding and the role of the funders for the present study and, if applicable, for the original study on which the present article is based |  | N/A |

*Give information separately for cases and controls in case-control studies and, if applicable, for exposed and unexposed groups in cohort and cross-sectional studies.

**Note:** An Explanation and Elaboration article discusses each checklist item and gives methodological background and published examples of transparent reporting. The STROBE checklist is best used in conjunction with this article (freely available on the Web sites of PLoS Medicine at http://www.plosmedicine.org/, Annals of Internal Medicine at http://www.annals.org/, and Epidemiology at http://www.epidem.com/). Information on the STROBE Initiative is available at www.strobe-statement.org.
