# Supplementary material for: A clinical prediction model to differentiate tuberculous spondylodiscitis from pyogenic spontaneous spondylodiscitis
Source: PLoS One. 2023 Aug 18;18(8):e0290361. doi: 10.1371/journal.pone.0290361 (PMC10437852; doi:10.1371/journal.pone.0290361)
Supplement: Attachment — Submitted filename: Response to Reviewer 2.8.23 PLOS ONE.docx [file pone.0290361.s004.docx]

**Response to Reviewer’s Comments**

**Response to Editor Comments**

The authors would like to thank the editor for your very valuable comment. We have addressed the issue that you raised below.

**Editor’s comment**

Data collection Lines 89-91

1. History suggestive of bacteremia (the presence of viable bacteria in the circulating blood with or without following symptoms: fever, chill, tachypnea, altered sensorium or hypotension, etc.) should be corrected to: “History suggestive of bacteremia e.g. fever and chill.”

History suggestive of bacteremia e.g. fever and chill.

**Response**: We have corrected “History suggestive of bacteremia (the presence of viable bacteria in the circulating blood with or without following symptoms: fever, chill, tachypnea, altered sensorium or hypotension, etc.)” to “History suggestive of bacteremia e.g. fever and chill.” as you suggested. (Page 4 line 89) It was highlight in gray.

Thank you very much for your help in improving this manuscript.
